# Whey Protein Supplementation Compared to Collagen Increases Blood Nesfatin Concentrations and Decreases Android Fat in Overweight Women: A Randomized Double-Blind Study

**DOI:** 10.3390/nu11092051

**Published:** 2019-09-02

**Authors:** Bruna M. Giglio, Raquel M. Schincaglia, Alexandre S. da Silva, Ieda C. S. Fazani, Paula A. Monteiro, João F. Mota, Juliana P. Cunha, Claude Pichard, Gustavo D. Pimentel

**Affiliations:** 1 Clinical and Sports Nutrition Research Laboratory (Labince), Faculty of Nutrition, Federal University of Goias, Goiânia 74605-080, Brazil; 2 Exercise and Immunometabolism Research Group, Postgraduation Program in Movement Sciences, Department of Physical Education, Universidade Estadual Paulista, Presidente Prudente, São Paulo 19060-900, Brazil; 3 Clinical Nutrition, Geneva University Hospital, 1205 Geneva, Switzerland

**Keywords:** obesity, whey protein, collagen, weight loss, leucine, amino acids

## Abstract

Protein supplements are usually used to control body weight, however, the impact of protein quality on body fat attenuation is unknown. We investigated the effects of isocaloric isoproteic supplementation of either whey protein (WG) or hydrolysed collagen supplementation (CG) on dietary intake, adiposity and biochemical markers in overweight women. Methods: In this randomized double-blind study, 37 women, [mean ± SE, age 40.6 ± 1.7 year; BMI (kg/m^2^) 30.9 ± 0.6], consumed sachets containing 40 g/day of concentrated whey protein (25 g total protein, 2.4 leucine, 1.0 valine, 1.5 isoleucine, *n* = 17) or 38 g/day of hydrolysed collagen (26 g total protein, 1.02 leucine, 0.91 valine, 0.53 isoleucine, *n* = 20) in the afternoon snack. The compliance was set at >70% of the total theoretical doses. The dietary intake was evaluated by a 6-day food record questionnaire. At the beginning and after eight weeks of follow-up, body composition was evaluated by using dual-energy X-ray absorptiometry and lipid profile, insulin resistance, C-reactive protein, adiponectin, leptin and nesfastin plasma concentrations were analyzed. Results: Supplements were isocaloric and isoproteic. There were no differences in caloric intake (*p* = 0.103), protein (*p* = 0.085), carbohydrate (*p* = 0.797) and lipids (*p* = 0.109) intakes. The branched chain amino acids (BCAA) (GC: 1.8 ± 0.1 g vs. WG: 5.5 ± 0.3 g, *p* < 0.001) and leucine intake (CG: 0.1 ± 0.1 g vs. WG: 2.6 ± 0.1 g, *p* < 0.001) were higher in WG compared to CG. BMI increased in the CG (0.2 ± 1.1 kg/m^2^, *p* = 0.044) but did not change in WG. WG decreased the android fat (−0.1 ± 0.3 kg, *p* = 0.031) and increased nesfatin concentrations (4.9 ± 3.2 ng/mL, *p* = 0.014) compared to CG. Conclusions: Whey protein supplementation in overweight women increased nesfatin concentrations and could promote increase of resting metabolic rate as part of body composition improvement programs compared to collagen supplementation for 8 weeks. Additionally, our findings suggest that collagen may not be an effective supplement for overweight women who are attempting to alter body composition.

## 1. Introduction

The expansion of adipose tissue, especially the visceral, causes chronic low grade inflammation which contributes to insulin resistance, [1] dyslipidaemia [2] and sarcopenic obesity [3]. Overweight individuals present changes in adipokines and hormones, such as adiponectin and nesfatin, which stimulate satiety and contribute to the reduction of long-term food intake [4,5,6]. Both overweight and obesity may also contribute to worsening an individual’s quality of life due to the physical overload of the additional weight [7].

As a reduction in weight can promote good health, innovative interventions are also necessary to reduce body weight. Protein supplements are investigated as a nutritional strategy to improve metabolic conditions and body composition [8]. Whey protein supplement contain peptides with antioxidant activities [9] and may increase satiety by stimulating anorexigenic hormones [10]. Whey protein is considered a high quality nutritional protein, mainly because it contains branched chain amino acids (BCAA), especially leucine, which contributes to the maintenance of muscle mass during weight loss [11]. BCAAs stimulate gut incretins secretion, which increase serum insulin concentrations. Some studies suggest that whey protein potentially has a therapeutic role in the reduction of glycemia in diabetic patients [12,13]. 

On the other hand, hydrolysed collagen, another widely used supplement, provides peptides that are absorbed into the small intestine. This protein source is generally regarded as having low biological value, mainly due to its low amounts of BCAA, lysine and tryptophan [14]. Recently, the consumption of hydrolysed collagen has been used in the prevention of lesions and tissues repairment [15], weight loss [16], fat free mass and muscle strength improvements [17]. However, there is little scientific evidence to substantiate the clinical use of collagen protein.

Due to a lack of prospective studies and controversial results comparing the effects of whey protein and hydrolysed collagen on body composition and the improvement of risk factors associated to those who are overweight, we hypothesized that whey protein concentrate compared to hydrolysed collagen supplementation reduces body fat, food intake and improves secretion of anorexigenic hormones in overweight women. This study aims at evaluating the effects of whey supplementation protein versus hydrolysed collagen on dietary intake, adiposity and biochemical markers in overweight women. 

## 2. Materials and Methods

### 2.1. Patients and Design of Study

A double-blind, randomized, eight-week clinical trial was conducted with fifty-two overweight women (20–58 years of age and body mass index (BMI) ≥ 25 kg/m^2^) that were assigned to either whey protein group (WG) or control group (CG) interventions. Exclusion criteria included women diagnosed with renal diseases, cardiovascular diseases, liver failure, cancer, those who were pregnant, lactating and polycystic ovary syndrome, chronic alcoholics or the use of anti-inflammatory drugs affecting appetite or body weight; in an inflammatory or infectious process on the day of collection, in the use of food supplements in the last six months and intolerant to lactose). The World Health Organisation (WHO) [18] showed a higher prevalence of obesity in women (22.9%) than men (17.2%), thus we decided to investigate only women. All testing was conducted at the Clinics Hospital and Faculty of Nutrition at the Federal University of Goiás between June 2016 and September 2017.

The study was approved by the Research Ethics Committee of the Federal University of Goiás, protocol number 1.470.285 and was registered at the Brazilian Registry of Clinical Trials (ReBEC) as RBR- 27 fmyt. Each patient was informed of the purpose of the study, experimental procedures and signed a consent form before their inclusion in the study. All procedures adopted are in accordance with the Declaration of Helsinki 1975, as revised in 1983.

### 2.2. Supplementation 

The patients were randomized into two groups to receive isocaloric isoproteic supplementation—Collagen group (CG) which received one sachet per day containing 38 g of supplement (144 kcal/day, 10 g of carbohydrate, 26 g of hydrolysed collagen, 2 g of vanilla flavouring and 0.001 g of sucralose, GELITA Bioactive Collagen Peptides^®^, German, lot H4501659); Whey group (WG) consumed one sachet per day containing 40 g of whey protein concentrate (160 kcal/day, 10 g of carbohydrate, 25 g of protein and 2.3 g of total fat, Maxtitanium^®^, Matão, Brazil, lot: 020745533400). Their contents appear in Supplementary Table S1. Randomization was conducted by an investigator who had no clinical involvement in the trial using www.randomizer.org. Patients were instructed to mix each package with 100 mL of water or juice and drink it as an afternoon snack once a day. All supplements had the same appearance, colour and taste; vanilla flavouring was added in the collagen group. Supplements were coded differently in each group (A or B) to blind the investigator. High protein diets including ~30 g protein per eating may help to improve appetite control and weight management, according to Phillips et al. [11]. 

Adherence to treatment was assessed by counting the number of supplements remaining when the participants returned to the laboratory. The value of 70% of the total consumption of the supplementation was adopted as criterion of adherence and patients who were below 70% of compliance were excluded. 

### 2.3. Palatability of Supplements 

In order to examine the palatability of supplements, patients were asked to answer a six point hedonic scale (1: extremely unpalatable; 2: moderately unpalatable; 3: neither unpalatable nor palatable; 4: slightly palatable; 5: moderately palatable; 6: extremely palatable), as previously described [19]. The palatability of the supplements assessed as ‘extremely unpalatable’ or ‘moderately unpalatable’ which could influence the compliance of supplement intake. It is also important to evaluate whether supplements have differences in taste that could negatively influence the results.

### 2.4. Food Intake

The patients were instructed to maintain their usual diet in order to verify if the supplement promoted changes in their food intake during the intervention period. The 24-h dietary recall was completed following the USDA’s Automated Multiple-Pass Method [20]. Compliance with the consumption of food was monitored through a 6-day food record questionnaire that included four days during the week and two days during the weekend. The calculation of total calories, macronutrients (proteins, carbohydrates, lipids and fibres), valine, isoleucine and leucine was performed using the Dietpro^®^ software (version 5.8, Minas Gerais, Viçosa, Brazil) by means of the American Food Composition Table (USDA).

### 2.5. Physical Activity Level

The International Physical Activity Questionnaire short version (IPAQ) validated for the Brazilian population [21] was used to evaluate the physical activity level of patients. Metabolic equivalent tasks (MET) were calculated from the data obtained. The patients were instructed to maintain their normal lifestyle habits. 

### 2.6. Body Composition

Body weight and height were measured using a digital scale (Filizola^®^, São Paulo, Brazil) with a precision of 0.1 kg and a stadiometer with precision in millimetres, respectively, for the BMI calculation. Body weight and height assessments were performed according to the procedures described by Lohman et al. [22]. The sagittal abdominal diameter (SAD) was measured to characterize the central adiposity of the volunteers [23]. Waist circumference was measured on undressed volunteers at the midpoint between the lower margin of the last palpable rib and the top of the iliac crest. Lean body mass, fat body mass, android and gynoid fat were assessed using the Dual-energy X-ray absorptiometry (DXA) technique (Lunar DPX NT—DXA for Bone Health—GE Healthcare^®^, Australia, New Zealand) [24].

### 2.7. Biochemical Parameters

Venous blood samples were collected into heparinized vacuum tubes at baseline and at the end of the 8-week intervention. After centrifugation at 4000 rpm for 10 min at 4 °C (Hitachi Koki^®^, Tokyo, Japan), plasma aliquots were stored at −80 °C (Panasonic MDF-U56VC-PA, Mexico City, Mexico) until analysis.

Glucose concentrations were determined by the enzymatic colorimetric method, high sensitivity C-reactive protein, using the chemiluminescence method and insulin, cholesterol, HDL-c, triacylglycerol by the immunoturbidimetry method (ArchitectPlus^®^, Naperville, IL, USA). HOMA-IR and HOMA-β were calculated to evaluate insulin resistance and functional capacity of pancreatic beta cells, respectively [25]. The fractions of low density cholesterol (LDL-c) and very low density cholesterol (LDL) were calculated according to those described by Friedewald et al. [26]. Leptin, adiponectin and nesfastin concentrations were assessed using the sensitive enzyme-linked immunosorbent assay (ELISA) kit (DuoSet, R&D systems^®^, Minneapolis, MN, USA) with assay range of 31.3 to 2000 pg/mL, 62.5 to 4000 pg/mL, 31.3 to 2000 pg/mL, respectively. 

### 2.8. Quality of Life

The quality of life was measured with the Short Form 36 Health Survey (SF-36). The SF-36 questionnaire consist of 36 items which are used to calculated eight subscales: physical activity, physical role, emotional role, vitality, mental health, social activity, pain and general health [27].

### 2.9. Statistical Analysis

Sample-size calculations, were based on a clinical trial study [28] for comparison means of reduction of adipose mass, two-tailed type, absolute error of 5%, effect size of 20% and test power of 80% that determined a sample of 20 patients for each group.

Normality was assessed with the Shapiro-Wilk test. The comparisons of means between groups, at the beginning of the study, in raw data or variations at baseline moment, were performed using the one-way ANOVA test. Changes in quality life and distribution of protein and BCAA in the meals were obtained by 2 factor ANOVA including the time (pre vs. post) as a repeated factor and supplement (whey protein compared with collagen) as an intra-group factor. The comparison of palatability of supplements was verified by the Wilcoxon test. Changes in body composition, biochemical makers and dietary intake were assessed by 2-factor ANCOVA with initial protein and fibre intake as a covariate, time (pre vs. post) as a repeated factor and supplement (whey protein compared with collagen) as an intra-group factor. Post-hoc comparisons were conducted using Sidak corrections. All analysis was conducted using the SPSS (IBM, Armonk, NY, USA) version 23. The level of significance was set 5% (*p* < 0.05). Unadjusted means and standard error (SE) are shown in the tables and text. 

## 3. Results

### 3.1. Patients

**Figure 1:**
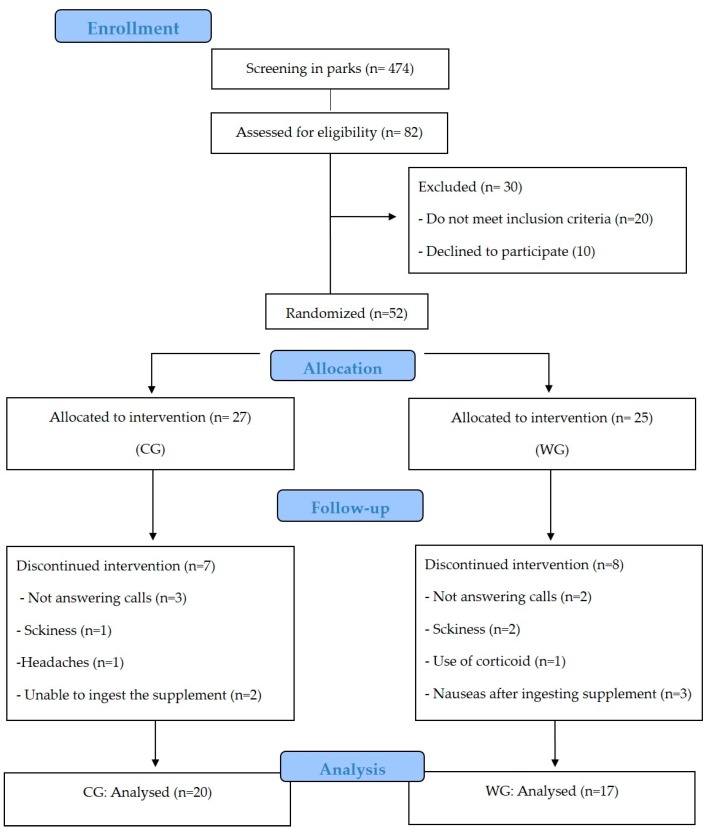
CONSORT flow diagram. CONSORT, Consolidated Standards of Reporting Trials. CG: collagen group, WG: whey protein group.

Out of 52 subjects included in the study, seven women (assigned to the CG) withdrew during the intervention: not answering calls (*n* = 3), sickness (1), headache (1), unable to ingest the supplement (2) and eight women (assigned to the WG): not answering calls (*n* = 2), sickness (2), use of corticoids (1), nausea after ingesting supplement (3). The analysis was performed only on those participants who completed the study with a compliance of supplement intake about 94% in WG and 92% in CG (*p* = 0.94) (Figure 1). The evaluation of baseline showed no difference between the WG and CG (Table 1). 

### 3.2. Palatability 

**Figure 2:**
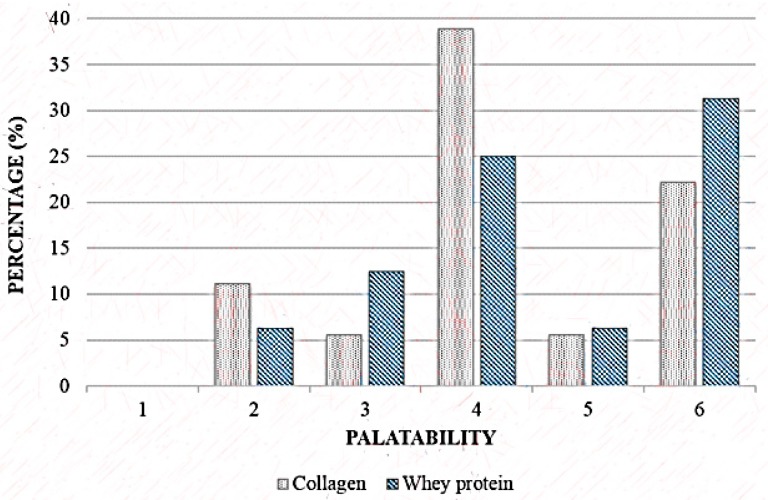
Palatability scale of Collagen and Whey protein group. 1: extremely unpalatable; 2: moderately unpalatable; 3: neither unpalatable nor palatable; 4: slightly palatable; 5: moderately palatable; 6: extremely palatable. Wilcoxon’s test, *p* = 0.61.

Among the volunteers who ingested the hydrolysed collagen, 38.4% judged the supplement neither unpalatable nor palatable and 31.3% declared the whey protein supplement moderately palatable, no significant difference between the groups (*p* = 0.640) (Figure 2). 

### 3.3. Food Intake

There was no difference between groups in protein (*p* = 0.069), lipids (*p* = 0.896), carbohydrates (*p* = 0.180), leucine (*p* = 0.276), BCAA (*p* = 0.222) and total calories (*p* = 0.677) nor intake at baseline. However, fibre and protein intake was higher in the CG compared to WG (*p* = 0.016) at baseline.

When the time x supplement interaction was evaluated, WG increased fibre intake by 6.6 ± 2.6 g during the study and 3.5 ± 1.8 g at week-8 compared to the baseline (*p* = 0.008) (Table 2). There were no differences in caloric intake (*p* = 0.103), proteins (*p* = 0.085), carbohydrates (*p* = 0.797) and lipids (*p* = 0.109).

**Figure 3:**
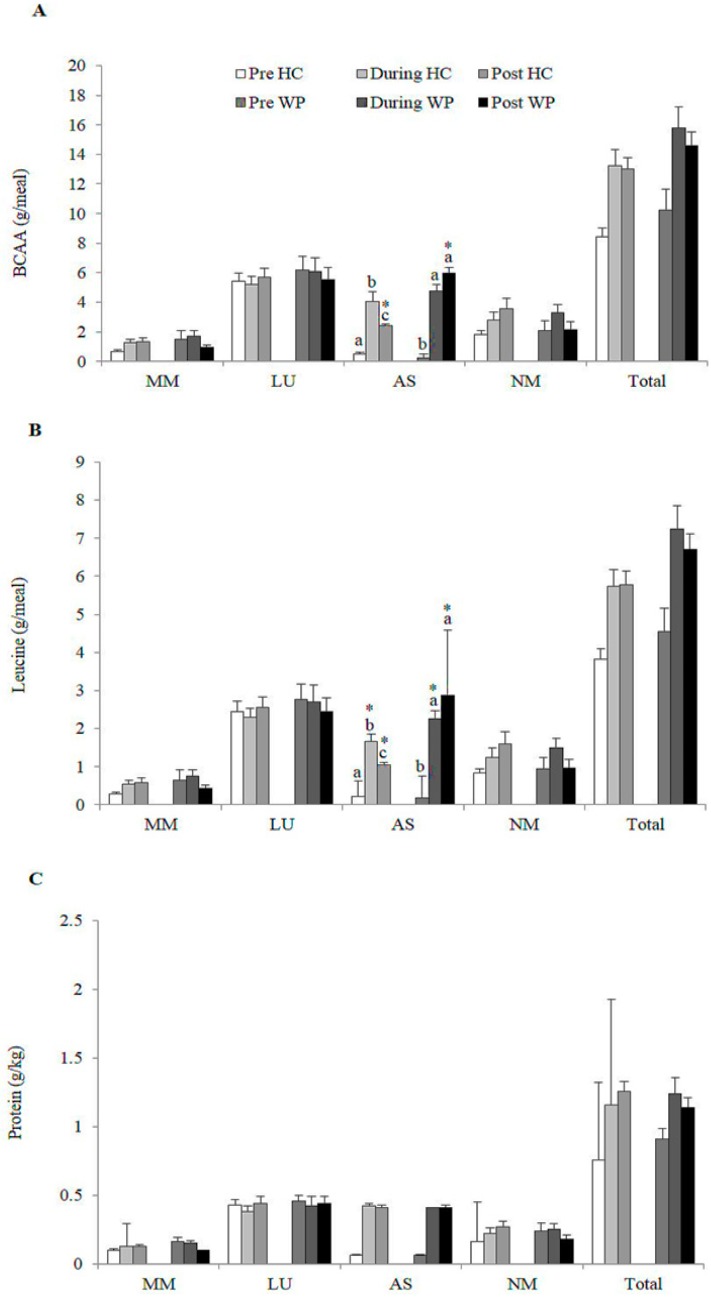
Distribution of total (**A**) Branched chain amino acid (BCAA) (g/meal); (**B**) Leucine (g/meal); (**C**) Protein (g/kg) consumption between meals performed by the participants of each group; HC: Hydrolysed collagen; WP: Whey protein; Afternoon snack; LU: Lunch; AS: Afternoon snack; MM: Morning meal; NM: Evening meal. Equal lowercase letters indicate means that do not differ statistically from one another by the Sidak test at Table 5. level of significance in the comparison between groups within each time. * indicate means that do not differ statistically from each other by the Sidak test at the 5% level of significance in the comparison between times within each group.

The intake of BCAA (CG: 1.8 ± 0.1 g vs. WG: 5.5 ± 0.3 g, *p* < 0.000) and leucine (CG: 0.1 ± 0.1 g vs. WG: 2.6 ± 0.1 g, *p* < 0.000) was higher in WG in the afternoon snack. There was no difference in protein intake (g/kg) between groups in the other meals and moments analysed (Figure 3).

### 3.4. Body Composition

After adjustments for the proteins and fibres intake, BMI increased in the CG (0.2 kg/m^2^, *p* = 0.044) compared to the baseline but did not differ among the groups. Android fat decreased in WG (−0.1 kg, *p* = 0.031) with difference between groups (Table 3). There was no difference in body mass, waist circumference, SAD and total lean body mass at the end of the intervention (*p* > 0.05).

### 3.5. Biochemical Parameters

Glucose concentrations, hs-CRP, insulin, HOMA-IR, HOMA-β, lipid profile, adiponectin and leptin adjustments did not change in the WG and CG groups. WG increased nesfatin concentrations compared to CG (4.9 ng/mL, *p* = 0.014) (Table 4). 

### 3.6. Quality of Life

Regarding questions found on the SF-36, such as physical activity (*p* = 0.987), physical role (*p* = 0.056), emotional role (*p* = 0.058), vitality (*p* = 0.636), mental health (*p* = 0.442), social activity (*p* = 0.963), pain (*p* = 0.596) and general health (*p* = 0.908) no difference was found between the supplementations (Table 5).

## 4. Discussion

The main finding of the present study was that eight-week supplementation with 40 g/day of whey protein in overweight women reduced the android fat and increased secretion of blood nesfatin. While in the collagen protein group, there was an increase in BMI, no changes in the other parameters were found.

In the present study, we found that protein consumption was around 1.2 g/kg/day. Likewise, other studies suggest that a high-protein diet (~1.2–1.6 g/kg/day) may be beneficial for weight loss, fat mass reduction and attenuation of muscle mass loss compared to normoprotein diet (~0.80 g/kg/day) [29,30] which is an important factor in the regulation of caloric intake and also in the control of obesity [31]. Furthermore, protein distribution (~25–30 g) per meal may contribute to improve appetite control, satiety and weight management [29]. In our study, both groups increased protein intake in the afternoon snack with 25 g protein supplementation, although the amount consumed of BCAAs and leucine in the WG was significantly higher when compared to the CG.

Whey protein is considered a high-quality protein because it contains BCAAs associated with satiety [32], regulation of food intake [33] and loss of lean body mass attenuation during caloric restriction [8]. An increase on protein quality intake in the afternoon snack may also have contributed to the reduction of android fat in WG. Similar results in overweight individuals were found by Frestedt et al. [34], which demonstrated that a mixture of whey protein isolate with other peptides had higher weight loss potential compared to those who consumed glycose. Furthermore, they also had greater reduction of body fat (6.1%) and a better control of lean body mass than the control group. Baer et al. [35] also demonstrated that the supplementation with 56 g of whey protein for 23 weeks reduced body weight and body fat compared with carbohydrates in overweight individuals. It is important to note that significant results were observed in studies that evaluated high-protein diets with energy restriction and showed greater reduction of body weight, fat mass and maintenance of lean body mass [30,36]. In the present study, caloric restriction was not part of the intervention and changes in body composition were small. However, we suggest that the habitual consumption of supplementary protein may contribute with the reduction of fat mass and long-term weight loss.

Collagen supplementation did not alter caloric intake but it increased body weight after eight weeks of intervention, possibly because the protein did not contain BCAA and tryptophan, which contribute to satiety and improvement of body composition [11]. Zdzieblik et al. [17] concluded that supplementation with 15 g/day of collagen in sarcopenic elderly in combination with resistance training for 12 weeks led to an extraordinary gain of fat free mass (4.2 ± 3.3 kg) and reduction fat mass (−5.5 ± 3.2 kg). Despite the promising results found by Zdzieblik [17], they are contradictory [37] and Moore et al. [38] demonstrated that 15 g/day with 0.4 g of leucine were not considered sufficient to promote protein synthesis. The difference in fat free mass between the collagen-supplemented group and the placebo group reported in the study was approximately 1.3 kg, being 2.7 to 5.6 times greater when compared to the values reported in the meta-analysis [39,40] that also evaluated the influence of protein supplementation and resistance exercise on body composition. In addition, Oikawa et al. [41] did not find benefits of collagen supplementation in elderly subjects to a period of energy restriction and physical inactivity.

We did not observe changes in the lean body mass at the end of our study. More significant changes in body composition and lean body mass may be observed if whey protein supplementation was combined with resistance training and adequate protein intake [8,42], justifying the lack of results in WG.

As well as insulin, nesfatin exerts an impact on glucose homeostasis, leading to the reduction of glucose levels but the mechanisms are not completely elucidated [43]. Nesfatin has been suggested as an anorectic hormone derived from the precursor peptide, nucleobindin 2 (NUCB2) that acts to increase satiety [44]. Peripheral tissues and the nervous system can stimulate their secretion. In addition, it has been demonstrated that nesfatin from peripheral organs can cross the blood-brain barrier through an unsaturated mechanism and be transported to the brain aiding in the regulation of appetite [45]. Bee et al. [46] suggest that in overweight individuals the efficiency of nesfatin absorption in the brain is reduced, possibly due to the saturation of the transporters of this hormone. However, Tsuchiya et al. [47] identified that overweight individuals may be deficient in the action of nesfatin and their increased plasma concentrations could contribute to the regulation of body weight. In addition, it has been reported that individuals with excess weight and with low concentrations of nesfatin tend to have an increase in total caloric intake [48]. In the present study, nesfatin concentrations increased twice after supplementation with whey protein but without modification to the caloric intake between groups, even though WG reduced android fat. As shown by Mirzaei et al. [48], nesfatin concentrations are associated with calorie, carbohydrate, protein intake and percentage fat in overweight patients, indicating a possible contribution to energy homeostasis. Since our study did find a correlation between delta of nesfatin and android fat (r = −0.21, *p* = 0.314), we cannot suggest that a reduction in body composition raises nesfatin concentrations. Therefore, based on the existence of few but conflicting studies, more studies are needed to understand the mechanisms of nesfatin on body composition.

### Study Limitations

The evaluation of dietary intake through the 24-h recall may have been underestimated at the start of the study since the CG volunteers reported consuming 19.04 kcal/kg and those of the GW 17.76 kcal/kg daily. It has already been reported that overweight women tend to under-report food intake [49]. However, it is possible to state that the protein intake was not affected, since the ingestion of the groups were similar to the end of the study and all the research data was adjusted for the initial value of proteins. In the present study, the groups were supplemented with protein and it was not possible to compare the results with a third group without supplementation. However, our main objective was to verify the effects of different animal protein sources among the volunteers. Comparing these sources, whey protein appears to have better results in overweight women than collagen. We suggest that further studies evaluate the intake of these supplements using a control group without supplementation and perform the resting metabolic rate evaluation. In addition, our intervention time was limited to eight weeks and better results can be observed with supplementation over a longer term supplementation. Additionally, as a woman ages she experiences a reduction in oestrogen which can affect her metabolic profile. We also suggest that future studies evaluate the influence of nesfatin metabolization in men to verify the implications of protein intake and concentrations of this hormone on body weight control. Additionally, a small sample size does not allow extrapolating our data for different demographics, such as men, obese women and those who regularly engage in physical activity. 

In conclusion, eight weeks of whey protein supplementation increased nesfatin concentrations and reduced the android fat compared to collagen. Whey protein supplementation could promote increase of resting metabolic rate as part of body composition improvement programs in overweight women. Therefore, our findings suggest that collagen may not been an effective supplement for overweight women who are attempting to alter body composition.

## Figures and Tables

**Table 1:** Baseline characteristics ^1^.

Variables	Collagen (*n* = 20)	Whey Protein (*n* = 17)	*p* ^2^
Age (years)	43 ± 1.8	37.8 ± 2.9	0.135
**Anthropometry**			
Body weight (kg)	79.9 ± 2.6	81.6 ± 3.5	0.694
Height (m)	160.5 ± 1.1	161.5 ± 1.3	0.549
BMI (kg/m^2^)	30.9 ± 0.8	31.1 ± 1	0.887
WC (cm)	92.5 ± 1.7	93 ± 2.1	0.839
SAD (cm)	26.6 ± 0.4	27.1 ± 0.6	0.531
**Body composition**			
Lean body mass (kg)	39.7 ± 1.4	40.5 ± 1.9	0.754
Lean body mass (%)	49.9 ± 1.1	49.8 ± 1.3	0.962
Fat body mass (kg)	37.4 ± 1.7	38.4 ± 2.1	0.725
Fat body mas (%)	48.3 ± 1.2	48.4 ± 1.3	0.950
Android fat (kg)	3.1 ± 0.1	3.3 ± 0.2	0.637
Gynoid fat (kg)	6.8 ± 0.3	7.1 ± 0.4	0.612
**Biochemical parameters**			
Hs-CRP (mg/dL)	6.3 ± 1.7	11.7 ± 2.9	0.109
Glucose (mg/dL)	90.9 ± 2.6	86 ± 1.4	0.136
Insulin (μU/mL)	11.7 ± 1.4	13.5 ± 1.6	0.408
HOMA-IR	2.7 ± 0.4	2.8 ± 0.3	0.820
HOMA-β	161.1 ± 17	227.7 ± 30.5	0.055
Cholesterol (mg/dL)	194.4 ± 9.1	179 ± 10.1	0.266
Triacylglycerol (mg/dL)	154.1 ± 20	160.2 ± 22	0.836
HDL-c (mg/dL)	57 ± 2.2	52 ± 2.6	0.155
LDL-c (mg/dL)	106.6 ± 7.5	95 ± 8.2	0.306
VLDL (mg/dL)	30.8 ± 4	32 ± 4.4	0.836
Adiponectin (μg/mL)	22 ± 0.2	22 ± 0.1	0.981
Leptin (ng/mL)	87 ± 4.7	79.2 ± 7	0.353
Nesfatin (ng/mL)	3.2 ± 0.5	6.9 ± 2.1	0.123
**Physical activity level**			
Met (min/week)	1122 ± 513.2	1284.1 ± 512.9	0.926

^1^ Values are means ± standard error. BMI: Body Mass Index; HDL: High-density lipoprotein cholesterol; HOMA-β: homeostatic model assessment of β cell function; HOMA-IR: homeostatic model assessment of insulin resistance; Hs-CRP: High sensitivity C-reactive protein; LDL-c: Low-density lipoprotein cholesterol; Met: Metabolic equivalents; SAD: Sagittal abdominal diameter; VLDL: very Low-density lipoprotein cholesterol; WC: Waist circumference. ^2^ *p* value obtained by one-way ANOVA test.

**Table 2:** Food intake ^1^.

Variables	Collagen (*n* = 20)			Whey Protein (*n* = 17)		*p* ^2^	
	Pre	During	Post	Pre	During	Post	
Calorie (kcal)	1522.6 ± 113.5	1610.6 ± 90.7	1696.9 ± 90.3	1450.1 ± 131.4	1939.1 ± 165.1	1712.4 ± 153.9	0.103
Carbohydrate (g)	174.3 ± 11.7	174.4 ± 10.2	188.1 ± 11.9	147 ± 16.6	230 ± 23.3	204.2 ± 24.9	0.797
Protein (g)	59.8 ± 3.5	93.5 ± 4.6	99.7 ± 4.9	74.1 ± 7.1	103.1 ± 7.9	91.5 ± 5.5	0.085
Lipids (g)	59.9 ± 6.8	58.1 ± 4.2	59.4 ± 3.9	61.1 ± 6.4	70.8 ± 9.0	60.3 ± 4.5	0.109
Fibre (g)	13.9 ± 1.1	14.1 ± 0.8	15.2 ± 1.5	10 ± 1 ^†‡^	16.6 ± 2	13.5 ± 1.8	0.008 *

^1^ Values are means ± standard error. ^2^ Calculated by 2- factor repeated-measures ANCOVA with fibres and proteins values in the initial moment as a covariate, * *p* < 0.05. ^†^ Difference between groups at the same moment by the Sidak test at the 5% level of significance. ^‡^ Difference between moment at the same group by the Sidak test at the 5% significance level in the comparison between times within each group.

**Table 3:** Body composition ^1^.

Variables	Collagen (*n* = 20)		Whey Protein (*n* = 17)		*p* ^2^
	Pre	Post	Pre	Post	
Body weight (kg)	79.9 ± 2.6	80.5 ± 2.6	81.6 ± 3.5	81.3 ± 3.5	0.089
BMI (kg/m^2^)	30.9 ± 0.8	31.2 ± 0.8 ^‡^	31.1 ± 1	31.0 ± 1 ^†^	0.044 *
WC (cm)	92.5 ± 1.7	92.2 ± 1.8	93.0 ± 2.1	92.2 ± 2.2	0.596
SAD (cm)	26.6 ± 0.4	26.3 ± 0.5	27.1 ± 0.6	26.8 ± 0.8	0.385
LBM (kg)	39.7 ± 1.4	40.2 ± 1.4	40.5 ± 1.8	41.0 ± 1.9	0.982
LBM (%)	49.9 ± 1.1	50.1 ± 1	49.8 ± 1.3	50.7 ± 1.3	0.370
BF (kg)	37.4 ± 1.7	37.6 ± 1.6	38.4 ± 2.1	37.6 ± 2.1	0.157
FBF (%)	48.3 ± 1.2	48.1 ± 1.1	48.4 ± 1.3	47.5 ± 1.3	0.345
Android fat (kg)	3.1 ± 0.1	3.2 ± 0.1	3.3 ± 0.2	3.1 ± 0.2 ^† ‡^	0.031 *
Gynoid fat (kg)	6.8 ± 0.3	6.9 ± 0.4	7.1 ± 0.4	7 ± 0.4	0.092

^1^ Values are means ± standard error. BMI: Body mass index; BF: body fat; LBM: lean body mass; SAD: Sagittal abdominal diameter; WC: waist circumference. ^2^ Calculated by 2-factor repeated-measures ANCOVA with fibres and proteins values in the initial moment as a covariate, *p* < 0.05. ^†^ Difference between groups at the same moment by the Sidak test at the 5% level of significance. ^‡^ Difference between moment at the same group by the Sidak test at the 5% significance level in the comparison between times within each group. * *p* < 0.05 was considered as significant.

**Table 4:** Biochemical parameters ^1^.

Variables	Collagen (*n* = 20)		Whey Protein (*n* = 17)		*p* ^2^
	Pre	Post	Pre	Post	
Hs-CRP (mg/dL)	6.3 ± 1.7	7.6 ± 2.1	11.7 ± 2.8	9.7 ± 2	0.244
Glucose (mg/dL)	90.9 ± 2.6	90.4 ± 2.5	86 ± 1.4	83 ± 1.6	0.512
Insulin (μU/mL)	11.7 ± 1.4	12.4 ± 1.5	13.5 ± 1.6	10.3 ± 1.3	0.095
HOMA-IR	2.7 ± 0.4	2.8 ± 0.4	2.8 ± 0.3	2.1 ± 0.2	0.094
HOMA-β	161.1 ± 17	169.9 ± 15.4	227.7 ± 30.5	229.8 ± 52.2	0.807
Cholesterol (mg/dL)	194.4 ± 9.1	188.5 ± 10.3	179 ± 10.1	186 ± 12.8	0.133
Triacylglycerol (mg/dL)	154.1 ± 20	161.1 ± 21	160.2 ± 22	132.7 ± 14	0.135
HDL-c (mg/dL)	57 ± 2.2	55.7 ± 1.9	52 ± 2.6	55.4 ± 3.1	0.179
LDL-c (mg/dL)	106.6 ± 7.5	100.5 ± 8.5	95 ± 8.2	103.9 ± 10.5	0.051
VLDL (mg/dL)	30.8 ± 4	32.2 ± 4.2	32 ± 4.4	26.5 ± 2.8	0.135
Adiponectin (μg/mL)	22 ± 0.2	21.2 ± 1.0	22 ± 0.1	22.1 ± 0.1	0.747
Leptin (ng/mL)	86.3 ± 4.7	87.4 ± 5.1	79.2 ± 7	93.2 ± 4.2	0.128
Nesfatin (ng/mL)	3.5 ± 0.4	3.6 ± 0.5	6.9 ± 2.1	12.6 ± 2.4 ^†‡^	0.014 *

^1^ Values are means ± standard error. Hs- CRP: Ultra-sensitive C-reactive protein; HDL: high density lipoprotein cholesterol; HOMA-β: homeostatic model assessment of β cell function; HOMA-IR: homeostatic model assessment of insulin resistance; LDL-c: low density lipoprotein cholesterol; VLDL: very low density lipoprotein cholesterol. ^2^ Calculated by 2- factor repeated-measures ANCOVA with fibres and proteins values in the initial moment as a covariate, *p* < 0.05. ^†^ Difference between groups at the same moment by the Sidak test at the 5% level of significance. ^‡^ Difference between moment at the same group by the Sidak test at the 5% significance level in the comparison between times within each group. * *p* < 0.05 was considered as significant.

**Table 5:** Quality of life ^1^.

Variables	Collagen (*n* = 20)		Whey Protein (*n* = 17)		*p* ^2^
	Pre	Post	Pre	Post	
Physical activity	83.5 ± 4.4	85 ± 3	79.4 ± 3.9	84.1 ± 3.5	0.505
Physical role	73.7 ± 6.6	85 ± 6.3	86.7 ± 5.7	82.3 ± 6.3	0.167
Emotional role	75 ± 8.6	96.6 ± 3.3	80.3 ± 6.4	78.4 ± 9	0.108
Vitality	61.5 ± 5.6	68 ± 5.3	59.1 ± 6.6	70.5 ± 4.8	0.424
Mental health	66 ± 6.8	73.6 ± 5.2	66.3 ± 6.3	77.8 ± 4.3	0.425
Social activity	75 ± 6.2	82.5 ± 5	74.2 ± 6.6	86.0 ± 4.7	0.570
Pain	64.3 ± 6.8	72.3 ± 5.7	65.4 ± 4.7	80.1 ± 5.2	0.386
General health	73.3 ± 4.6	76.1 ± 4.2	71.3 ± 6.2	73.2 ± 5.6	0.868

^1^ Values are means ± standard error. ^2^ Obtained by the repeated measures 2 factor ANOVA test to evaluate the time/supplement interaction.

## Supplementary Materials

The following are available online at https://www.mdpi.com/2072-6643/11/9/2051/s1, Table S1: Composition of protein supplements.
