# Supplementary material for: Whey Protein Supplementation Compared to Collagen Increases Blood Nesfatin Concentrations and Decreases Android Fat in Overweight Women: A Randomized Double-Blind Study
Source: Nutrients. 2019 Sep 2;11(9):2051. doi: 10.3390/nu11092051 (PMC6770102; doi:10.3390/nu11092051)
Supplement: Supplementary file 1 [file nutrients-11-02051-s001.pdf]

Supplementary Table 1. Composition of protein supplements

	CG supplement		WG supplement	
	**g/100g**	**g/38g**	**g/100g**	**g/40g**
Calorie	378.9	144	400	160
Carbohydrate	26.3	10	25	10
Lipids	0	0	5.7	2.3
Protein	68.4	26	62.5	25
Alanine	8.6	3.2	3	1.2
Arginine	7.3	2.7	2.5	1.0
Aspartic acid	5.8	2.2	6.2	2.5
Cystine	0	0	1.5	0.6
Glutamic acid	10.2	3.8	9.7	3.9
Glycine	22.2	8.4	1.2	0.5
Histidine	1.0	0.3	2.2	0.9
Hidroxiprolina	11.9	4.5	0	0
Hidroxilisina	1.6	0.6	0	0
Isoleucine	1.4	0.5	3.7	1.5
Leucine	2.7	1	6	2.4
Lysine	3.6	1.3	5.2	2.1
Methionine	0.9	0.3	1	0.4
Phenylalanine	2.1	0.7	1.7	0.7
Proline	12.7	4.8	4	1.6
Serine	3.2	1.2	3.7	1.5
Tyrosine	0.8	0.3	2.2	0.9
Tryptophan	0	0	1.7	0.7
Threonine	1.8	0.6	4	1.6
Valine	2.4	0.9	2.5	1.0

CG: Collagen group; WG: Whey protein group
